# Protein profiling of hemolymph in *Haemaphysalis flava* ticks

**DOI:** 10.1186/s13071-022-05287-7

**Published:** 2022-05-24

**Authors:** Lei Liu, Fen Yan, Lu Zhang, Zhi-feng Wu, De-yong Duan, Tian-yin Cheng

**Affiliations:** Research Center for Parasites & Vectors (RCPV), College of Veterinary Medicine, Hunan Agricultural University, Changsha, 410128 China

**Keywords:** Tick, Hemolymph, Enzyme, Enzyme inhibitors, Vitellogenin, Serpin, Cystatin, Microplusin

## Abstract

**Background:**

Tick hemolymph bathes internal organs, acts as an exchange medium for nutrients and cellular metabolites, and offers protection against pathogens. Hemolymph is abundant in proteins. However, there has been limited integrated protein analysis in tick hemolymph thus far. Moreover, there are difficulties in differentiating tick-derived proteins from the host source. The aim of this study was to profile the tick/host protein components in the hemolymph of *Haemaphysalis flava*.

**Methods:**

Hemolymph from adult engorged *H. flava* females was collected by leg amputation from the *Erinaceus europaeus* host. Hemolymph proteins were extracted by a filter-aided sample preparation protocol, digested by trypsin, and assayed by liquid chromatography–tandem mass spectrometry (LC–MS/MS). MS raw data were searched against the UniProt Erinaceidae database and *H. flava* protein database for host- and tick-derived protein identification. Protein abundance was further quantified by intensity-based absolute quantification (iBAQ).

**Results:**

Proteins extracted from hemolymph unevenly varied in size with intense bands between 100 and 130 kDa. In total, 312 proteins were identified in the present study. Therein 40 proteins were identified to be host-derived proteins, of which 18 were high-confidence proteins. Top 10 abundant host-derived proteins included hemoglobin subunit-α and subunit-β, albumin, serotransferrin-like, ubiquitin-like, haptoglobin, α-1-antitrypsin-like protein, histone H2B, apolipoprotein A-I, and C3-β. In contrast, 169 were high-confidence tick-derived proteins. These proteins were classified into six categories based on reported functions in ticks, i.e., enzymes, enzyme inhibitors, transporters, immune-related proteins, muscle proteins, and heat shock proteins. The abundance of Vg, microplusin and α-2-macroglobulin was the highest among tick-derived proteins as indicated by iBAQ.

**Conclusions:**

Numerous tick- and host-derived proteins were identified in hemolymph. The protein profile of *H. flava* hemolymph revealed a sophisticated protein system in the physiological processes of anticoagulation, digestion of blood meal, and innate immunity. More investigations are needed to characterize tick-derived proteins in hemolymph.

**Graphical Abstract:**

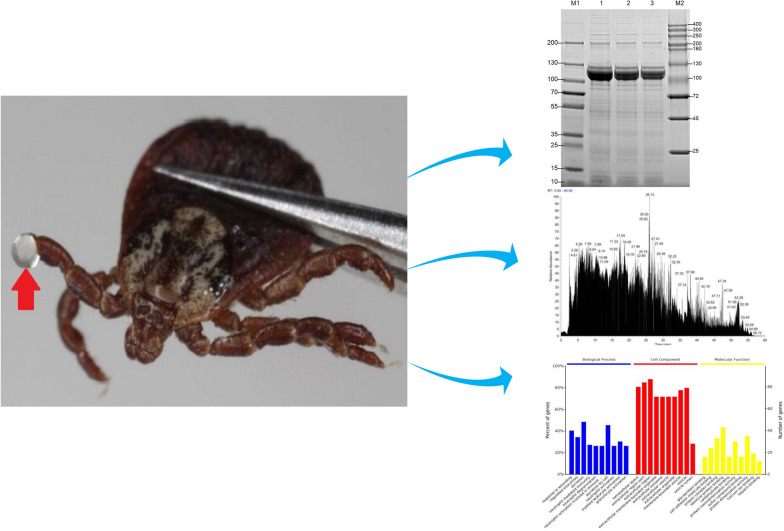

**Supplementary Information:**

The online version contains supplementary material available at 10.1186/s13071-022-05287-7.

## Background

Tick hemolymph is a circulating fluid that fills the body cavity and bathes the inner organs. It serves as an exchange medium for the transport of nutrients, hormones, and products of cellular metabolism, and offers protection against pathogens to which ticks are exposed [1]. Hemolymph consists of hemocytes and plasma. Plasma predominates in tick hemolymph, representing approximately 90% by weight.

In tick plasma, proteins are the most soluble components (11.5–14.3% by weight) [2]. Thus far, a few proteins have been isolated, identified, and partially characterized in tick hemolymph, including vitellogenins (Vgs) [3, 4], macroglobulins [5], antimicrobial peptides [6], defensins [7], lectins [8], carrier proteins [9, 10], myeloid differentiation-2-related lipid-recognition domain [11], serine proteinase inhibitor (serpin) [12], and protein disulfide isomerases [13]. However, owing to the tiny volume of hemolymph from ticks, it is difficult to characterize proteins in hemolymph using traditional protein chemistry methods. The limited and unsystematic information obstructs our understanding of the roles of hemolymph in tick development, reproduction, endocrine function, and defense against pathogens; it also hinders the discovery of new targets against ticks [14].

Proteomic approaches are efficient tools for mapping protein profiles in ticks. Madden et al. initially reported saliva protein profiles of two related tick species, *Amblyomma americanum* and *Amblyomma maculatum*, by matrix-assisted laser desorption/ionization–time-of-flight mass spectrometry (MALDI-TOF MS) [15]. Since then, proteomic investigations have been performed in *Ixodes scapularis* (saliva) [16], *Ornithodoros moubata* and *Ornithodoros erraticus* (salivary proteins) [17], *Rhipicephalus sanguineus* (saliva) [18], *Haemaphysalis flava* (fecal proteins and midgut contents) [19, 20], and *Rhipicephalus microplus* (saliva) [21]. Nevertheless, thus far there have been only two reports describing the protein profile in tick hemolymph. Stopforth et al. conducted a proteomic study to identify proteins secreted in the hemolymph of *Ornithodoros savignyi* ticks following immune challenge with yeasts [22]. Aguilar-Díaz et al. compared hemolymph proteomes of two *R. microplus* strains with different degrees of resistance to ixodicides [23]. Because of the lack of a transcriptome library of the tested ticks at the time, the number of hemolymph proteins identified in both studies was quite low.

In this study, hemolymph was collected from adult *H. flava* females. Proteins contained in the hemolymph were analyzed by liquid chromatography–tandem MS (LC–MS/MS) in combination with a search against the UniProt database and self-constructed *H. flava* transcriptome library, aiming to provide the most comprehensive data to data regarding host- and tick-derived proteins in tick hemolymph.

## Methods

### Collection of tick hemolymph

All experimental procedures were approved and overseen by the Institutional Animal Care and Use Committee at Hunan Agricultural University, with approval no. 2021085. Fully engorged *H. flava* ticks were picked from naturally infected hedgehogs in our experimental and observation station located in Xinyang City, Henan Province, China (31°44′N, 114°10′E). Hedgehogs, which are common hosts of *H. flava* ticks [24], were housed with no recent exposure to any chemical acaricides. Hemolymph from the ticks was collected according to a previous study [25]. Briefly, 45 engorged adult *H. flava* females were randomly selected, rinsed with water, and sterilized with 70% ethanol. Ticks were immobilized on Petri dishes with their ventral sides up using double-sided tape. The legs were cut off with ophthalmic scissors. Then, gentle pressure was applied to the tick body, and hemolymph was collected using a glass-capillary tube with 2 μl of protease inhibitor cocktail [26]. Hemolymph from 15 ticks was pooled to ensure adequate size for further analysis. Thus, these 45 ticks represent three replicates. The pooled hemolymph sample was transferred into a clean tube and centrifuged at 14,000×*g* for 10 min at 4 °C. Supernatant was collected and quantified with a Bradford Protein Assay Kit (Beyotime Biotechnology, Shanghai, China).

### Sodium dodecyl sulfate–polyacrylamide gel electrophoresis

Ten microliters of supernatant was mixed with SDT buffer (30 μl, 4% sodium dodecyl sulfate, 100 mM dithiothreitol, and 150 mM Tris–HCl pH 8.0). The mixture was subjected to a boiling-water bath for 5 min. After cooling to room temperature and centrifuging at 14,000×*g* for 10 min at 4 °C, samples were analyzed by sodium dodecyl sulfate–polyacrylamide gel electrophoresis (SDS-PAGE) with omniPAGE™ precast gels (LK204, 4–15%, Epizyme Biomedical Technology, Shanghai, China).

### Protein digestion by filter-aided sample preparation

We followed a filter-aided sample preparation protocol before LC–MS/MS analysis [27]. An aliquot of 20 μl supernatant was added to 5 μl 200 mM dithiothreitol, boiled in water for 5 min, and cooled to room temperature. Next, 200 μl 8 M urea buffer was introduced and mixed well. The mixture was transferred into an ultrafiltration tube fitted with a 10 kDa centrifugal filter unit, and centrifuged at 14,000×*g* for 15 min. Proteins retained on the filter were washed several times with 8 M urea buffer to ensure maximal removal of impurities. They were then mixed with 100 μl iodoacetamide solution, shaken at 600 rpm for 1 min, kept away from light at room temperature for 30 min, and then centrifuged at 14,000×*g* for 10 min. Proteins on the filter were rinsed twice with 8 M urea buffer and then incubated with 40 μl trypsin solution (3 μg trypsin in 40 μl 25 mM NH_4_HCO_3_, Sigma-Aldrich, MO, USA) at 37 °C for 16–18 h. Then, the centrifugal filter unit with digests on it was inserted into a new collection tube and centrifuged at 14,000×*g* for 10 min. Filtrates were collected and submitted to a C18 cartridge (Empore™ solid-phase extraction (SPE) C18 cartridges, bed I.D. 7 mm, volume 3 ml; Sigma-Aldrich, St. Louis, MO, USA) for desalination. Then they were concentrated by vacuum centrifugation and reconstituted in 40 µl of 0.1% (v/v) trifluoroacetic acid.

### Analysis by LC–MS/MS

LC–MS/MS analyses were performed on a Q Exactive mass spectrometer coupled to an EASY-nLC system (Thermo Fisher Scientific, Waltham, MA, USA). A total of 5 μg of peptides was injected. Peptides were passed through a C18 reversed-phase column (Thermo Scientific EASY-Column, 10 cm, 75 μm inner diameter, 3 μm resin) in buffer A (2% acetonitrile and 0.1% formic acid) and separated with a linear gradient of buffer B (80% acetonitrile and 0.1% formic acid) at a flow rate of 250 nl/min controlled by IntelliFlow technology over 60 min.

MS data were acquired using a data-dependent top 10 method dynamically choosing the most abundant precursor ions from the survey scan (300–1800 m/z) for higher-energy collisional dissociation (HCD) fragmentation. The target value was determined based on predictive automatic gain control (gAGC). The dynamic exclusion duration was set at 25 s. Survey scans were acquired at a resolution of 70,000 at m/z 200. The resolution for HCD spectra was set to 17,500 at m/z 200. The normalized collision energy was 30 eV. The underfill ratio was defined as 0.1%.

### Sequence database search and data processing

MS data were processed by MaxQuant software (version 1.6.1.0., https://maxquant.net/maxquant/). The MS/MS raw files were searched against the UniProt Erinaceidae database (28,253 entries, downloaded on 03/01/2021) for the identification of host proteins, and then against a *H. flava* protein database constructed in parallel with the transcriptome (https://www.ncbi.nlm.nih.gov/bioproject/PRJNA756707/) for identification of tick proteins, which contained 57,024 clusters and 10,859 predicted proteins. An initial search was set at a precursor mass window of 6 parts per million (ppm). The search followed an enzymatic cleavage rule of trypsin/P, and allowed a maximum of two missed cleavage sites and a mass tolerance of 20 ppm for fragment ions. Carbamidomethylation of cysteines was defined as fixed modification, while protein N-terminal acetylation and methionine oxidation were defined as variable modification. The cutoff for the global false discovery rate for peptide and protein identification was set to 0.01. Intensity-based absolute quantification (iBAQ) was carried out in MaxQuant.

## Results and discussion

### SDS-PAGE for total proteins in tick hemolymph

**Fig. 1:**
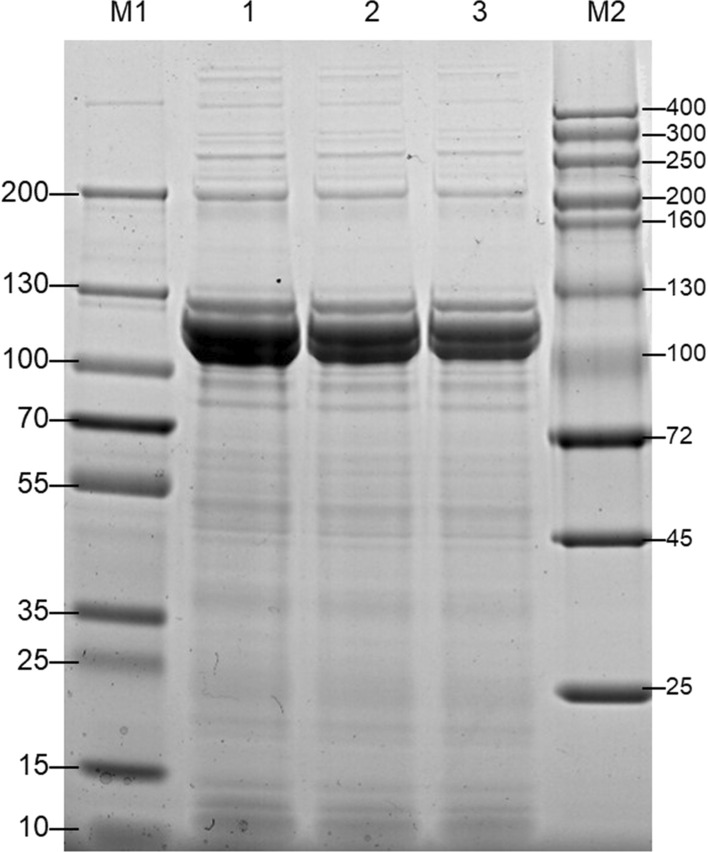
Sodium dodecyl sulfate–polyacrylamide gel electrophoresis analysis of proteins from *Haemaphysalis flava* hemolymph. M1, M2 = markers (kDa); 1, 2, 3 = three replicates of proteins from the *H. flava* hemolymph

The concentration of protein in tick hemolymph was determined to be 5.03 ± 0.19 μg/μl. Figure 1 presents an SDS-PAGE image of total hemolymph proteins. The electrophoretogram indicated that proteins in *H. flava* hemolymph varied greatly in size, with intense bands at 100–130 kDa.

Tatchell et al. firstly reported that hemolymph proteins from female *R. microplus* immediately after engorgement revealed 15 bands by SDS-PAGE, but 14 bands in the case of ovipositing females [28]. Thereafter, investigations of hemolymph proteins were undertaken in *O. moubata* [4], *Ornithodoros parkeri* [29], *Dermacentor variabilis* [30–32], and *Haemaphysalis longicornis* [33] using SDS-PAGE and native PAGE.

Protein components in hemolymph vary with tick species, and also display dynamic changes in various physiological processes. Stopforth et al. showed the size of hemolymph proteins of *O. savignyi* in the range of 14–200 kDa [22], but Boldbaatar et al. demonstrated that some hemolymph proteins in *H. longicornis* could be as large as 669 kDa [33]. Protein concentration and composition changed greatly in the hemolymph of female *O. parkeri* during blood-feeding [29]. Hefnawy revealed that the total content of hemolymph varied according to life stage and engorgement level [34].

### Host proteins in tick hemolymph

A search of the UniProt Erinaceidae database identified a total of 40 host proteins. Among these, 18 belonged to high-confidence proteins (unique peptides ≥ 2, Table 1). Of the 18 high-confidence proteins, 12 were components from host plasma, namely, albumin, ubiquitin-like, serotransferrin-like, α-1-antitrypsin-like protein, α-2-macroglobulin-like, fibrinogen α/β/γ chain, haptoglobin, C3-β, hemopexin, and apolipoprotein A-I. The other six proteins were from host blood cells, including hemoglobin (Hb) subunit-α/β, tubulin, histone H2B, carbonic anhydrase, and HSP90α.

**Table 1:** High-confidence proteins from the host (*Erinaceus europaeus*) identified in hemolymph of *Haemaphysalis flava* ticks

Protein overview	No. of unique peptides	Coverage (%)	Identity (%)	iBAQ (× 10^6^)
P01949, hemoglobin subunit α, *Erinaceus europaeus*	8	64.5	64.5	271.90 ± 59.13
A0A1S3WPY1, hemoglobin subunit β, *E. europaeus*	11	74.8	74.8	257.22 ± 55.55
A0A1S2ZRW6, serum albumin, *E. europaeus*	38	65.8	65.8	112.09 ± 27.87
A0A1S3W2S7, serotransferrin-like, *E. europaeus*	33	59.3	59.3	17.93 ± 4.42
A0A1S3W634, ubiquitin-like, *E. europaeus*	2	32.5	32.5	17.77 ± 4.71
A0A1S3WFY0, haptoglobin, *E. europaeus*	9	29.5	29.5	8.28 ± 2.12
A0A1S3WJ22, α-1-antitrypsin-like protein, *E. europaeus*	2	20.4	7	3.86 ± 0.87
A0A1S3WR78, histone H2B, *E. europaeus*	2	20.8	20.8	2.52 ± 0.63
Q9TS49, apolipoprotein A-I, *E. europaeus*	3	13.7	13.7	2.21 ± 0.68
A0A1S3WDL1, C3-β, *E. europaeus*	6	14.7	5.8	1.84 ± 0.51
A0A1S3W909, tubulin β chain, *E. europaeus*	5	15.9	15.9	1.77 ± 0.37
A0A1S3WJA6, fibrinogen γ chain, *E. europaeus*	4	14.5	14.5	1.46 ± 0.38
A0A1S3W2L2, α-2-macroglobulin-like, *E. europaeus*	13	12.4	12.4	1.34 ± 0.38
A0A1S3AIG2, carbonic anhydrase, *E. europaeus*	3	11.5	11.5	1.21 ± 0.30
A0A1S3A1I2, hemopexin, *E. europaeus*	3	8.4	8.4	0.86 ± 0.11
A0A1S2ZF33, HSP90-α, *E. europaeus*	4	7.1	7.1	0.80 ± 0.12
A0A1S3A559, fibrinogen α chain, *E. europaeus*	4	6.5	6.5	0.74 ± 0.21
A0A1S3WJK5, fibrinogen β chain, *E. europaeus*	4	8.6	8.6	0.71 ± 0.37

Host serum constituents have been detected in tick hemolymph, including Hb hydrolyzed fragments, immunoglobulin G (IgG), transferrin, and albumin [35]. However, the full spectrum of host proteins that could be transferred to tick hemolymph remained unknown [36]. Our data demonstrated that at least these 40 host plasma proteins could be transferred into tick hemolymph.

Mammalian fibrinogen is composed of two identical subunits, each subunit containing one α, β, and γ chain. Our data indicated the presence of a considerable number of host fibrinogen α, β, and γ chains in hemolymph, but did not detect any fibrinogen of tick origin. This observation implies that host fibrinogen was transferred intact from the midgut to the hemolymph. It is possible that the molecules and mechanisms involved in coagulation in tick hemolymph are the same as those in the host blood. In other words, ticks may share the same coagulation machinery as the hosts. Consistent with this assumption, anticoagulants used during the collection of tick hemolymph were protease inhibitor cocktail and ethylenediamine tetraacetic acid (EDTA) [26, 37]. The former inhibited serine protease, cysteine protease, aspartic protease, metalloprotease, and aminopeptidase, whereas the latter prevented blood from clotting by Ca^2+^ chelation.

### Tick-derived proteins in hemolymph

In total, 1196 unique peptides and 312 deduced protein sequences were identified by searching the *H. flava* transcriptome database (Additional file 1: Table S1). Among these tick sequences, 175 were high-confidence-deducing sequences (unique peptides ≥ 2) and belonged to 169 proteins, as several peptides were from the same protein. For instance, α2-macroglobulin-like protein covered peptides Cl-k.18726, Cl-k.14664, Cl-k.14873 and Cl-k.18058. No albumin of tick origin was detected.

**Fig. 2:**
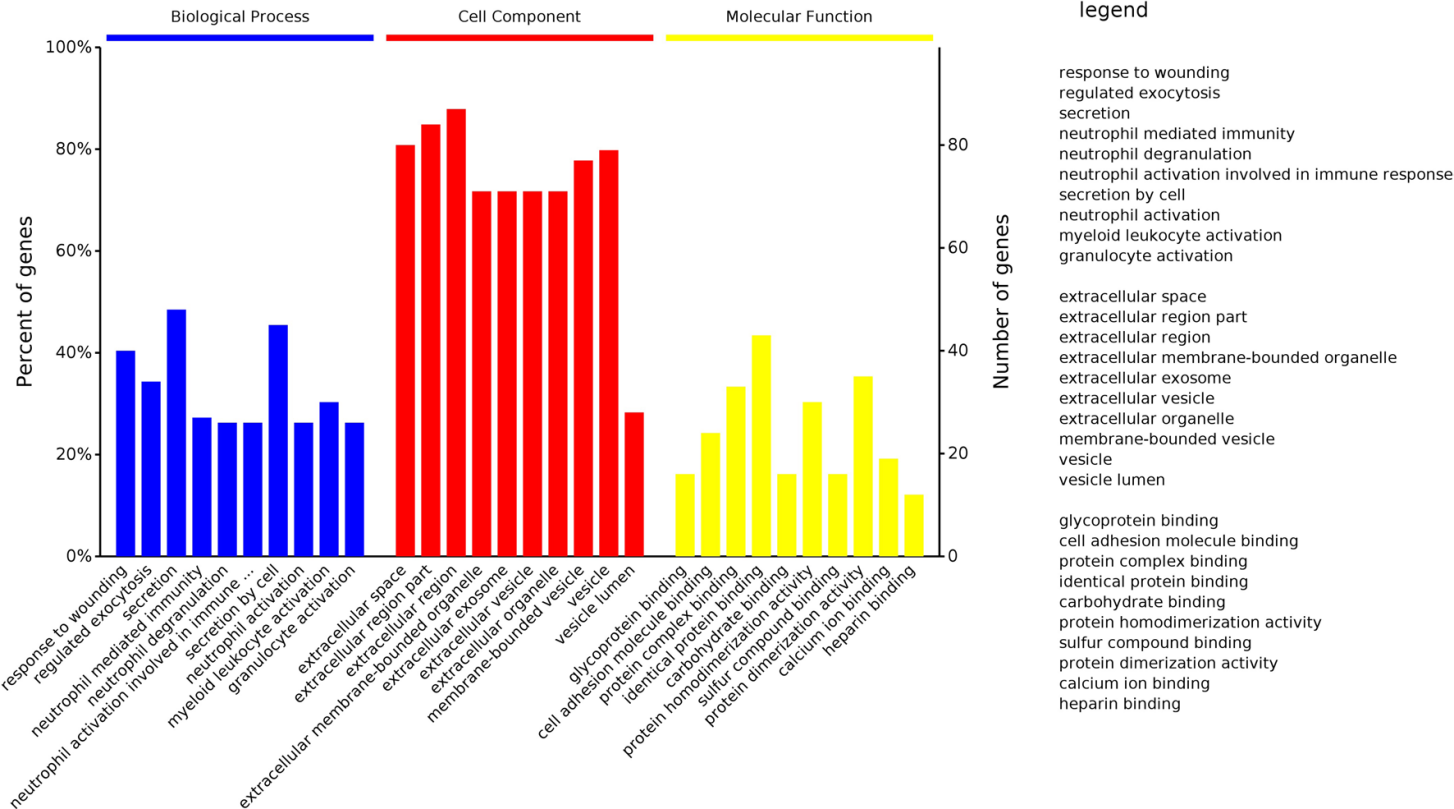
GO analysis of tick-derived proteins in hemolymph. The top ten GO terms of each category are as indicated

Gene Ontology (GO) analysis using OmicsBean (http://www.omicsbean.cn/) revealed that these 169 proteins were mainly enriched in biological processes of neutrophil, leukocyte, and granulocyte activation, and were significantly located in the extracellular space. Their molecular functions mainly included binding of proteins, carbohydrates, and other molecules such as sulfur compounds and calcium. The top ten GO terms of each category are displayed in Fig. 2.

We searched the literature in PubMed and the Chinese National Knowledge Infrastructure (CNKI), and found that 76 homologues of 169 high-confidence proteins were studied in the literature. Based on the conclusions of studies, these homologues were classified into six categories, including enzymes, enzyme inhibitors, transporters, immune-related proteins, muscle proteins, and others. Among them, enzymes were the most abundant. Their substrates included proteins, lipids, carbohydrates, and chitins. In addition, there were many types of serine proteases and their inhibitors (serpins).

Though some tick-derived proteins in hemolymph and other tissues have been characterized, the number of characterized proteins is relatively low compared to the total number of proteins detected in hemolymph. Hence, the functions of the majority of tick-derived proteins in hemolymph are as yet unknown, making it impossible to classify all of them based on function. The major tick-derived proteins in hemolymph with known functions will be discussed below.

### Enzymes in tick hemolymph

Although just a portion of enzymes are listed in Table 2, it is clear that the enzyme composition in tick hemolymph was diverse and complex. These enzymes were mainly involved in anticoagulation, digestion of blood meal, and innate immunity. They also participated in substance metabolism and even molting.

**Table 2:** High-confidence tick proteins with reported functions in the hemolymph of *H. flava* ticks

No.	Protein		Alignment				iBAQ (× 10^6^)
	ID	Length (amino acids)	Entry and overview	*E* value	Score	Identity (%)	
I. Enzymes							
1	Cl-k.18156 ①	376	L7M876, tick serine protease, *Rhipicephalus pulchellus*	0	1897	84.6	5.96 ± 1.32
2	Cl-k.17502 ①	484	A0A131Z7A0, tick serine protease, *Rhipicephalus appendiculatus*	0	1451	67.8	0.39 ± 0.04
3	Cl-k.19217 ①	437	A0A1E1X8K7, serine protease, *Amblyomma aureolatum*	0	1907	85.1	2.13 ± 0.47
4	Cl-k.19341	348	A1IHG0, longipain, *H. longicornis*	0	1814	93.5	7.34 ± 1.74
5	Cl-k.18108	443	A0A097CK68, enolase, *H. flava*	0	2233	99.5	1.16 ± 0.35
6	Cl-k.14381	507	A0A6M2D6D9, serine carboxypeptidase, *R. microplus*	0	2115	83.6	0.49 ± 0.15
7	Cl-k.18093	164	A0A131XJQ8, metalloproteinase, *Hyalomma excavatum*	1.7E−91	686	75.0	(7) 221.26 ± 58.79
8	Cl-k.18993	398	Q2WFX6, aspartic protease, *H. longicornis*	0	1990	95.9	1.47 ± 0.34
9	Cl-k.7217	397	A0A034WWI5, heme-binding aspartic peptidase, *R. microplus*	0	1343	68.0	3.03 ± 0.93
10	Cl-k.14313	561	A0A1E1XAU4, cysteine proteinase, *A. aureolatum*	0	2602	84.4	5.24 ± 1.17
11	Cl-k.18480	326	A0A023FWK4, cathepsin L, *Amblyomma parvum*	0	1604	88.7	99.93 ± 26.58
12	Cl-k.24797	110	A0A023GJU1, cathepsin C, *Amblyomma triste*	2.6E−70	563	92.7	2.08 ± 0.83
13	Cl-k.18626	416	L7M0J1, phospholipase a2, *R. pulchellus*	0	1704	80.1	25.19 ± 5.41
14	Cl-k.30316 ②	566	Q9U6M8, carboxylic ester hydrolase, *R. microplus*	5.1E−171	1290	47.3	10.59 ± 2.67
15	Cl-k.24717 ②	564	A0A6M2CHI4, carboxylic ester hydrolase, *R. microplus*	0	1366	49.7	0.25 ± 0.05
16	Cl-k.18635	521	A5LHV9, disulfide isomerase, *H. longicornis*	0	2514	96.2	1.74 ± 0.43
17	Cl-k.17461	163	F2Z7L0, lysozyme, *H. longicornis*	3.1E−70	544	87.1	2.44 ± 0.86
18	Cl-k.17638	397	G8C7A0, lysosomal acid phosphatase, *H. longicornis*	0	1878	93.5	75.50 ± 18.32
19	Cl-k.18136	233	Q6JVN0, glutathione S-transferase, *H. longicornis*	3.5E−159	1144	93.7	1.74 ± 0.50
20	Cl-k.18835	526	A0A131YK94, superoxide dismutase (Cu–Zn), *R. appendiculatus*	0	1475	61.8	18.83 ± 4.63
21	Cl-k.19315 ③	316	A0A131YMH9, chitinase, *R. appendiculatus*	0	1777	85.6	1.46 ± 0.44
22	Cl-k.18448 ③	536	A0A023FND8, chitinase, *A. cajennense*	0	1919	80.3	21.40 ± 4.31
23	Cl-k.17863	367	A0A286R6W4, fructose-bisphosphate aldolase, *Dermacentor silvarum*	0	1796	94.2	2.26 ± 0.57
24	Cl-k.35642	331	A0A1E1X9Y4, alpha-L-fucosidase, *A. aureolatum*	0	1414	81.7	1.87 ± 0.38
25	Cl-k.18180	430	A0A2P1DPZ4, glyceraldehyde-3-phosphate dehydrogenase, *H. flava*	0	1730	100	2.37 ± 0.62
26	Cl-k.17933	549	A0A6M2CTD4, ATP synthase subunit beta, *R. microplus*	0	2628	96.3	0.39 ± 0.02
II. Enzyme inhibitors							
27	Cl-k.20245 ④	256	A0A5P8H6S1, serpin-a, *H. longicornis*	9.3E−97	753	61.9	55.88 ± 13.09
28	Cl-k.17714 ④	415	A0A5P8H6S1, serpin-a, *H. longicornis*	6.9E−169	1246	63.4	0.25 ± 0.24
29	Cl-k.16905 ④	143	A0A5P8H6S1, serpin-a, *H. longicornis*	6.4E−49	422	61.8	32.01 ± 6.67
30	Cl-k.18212 ④	427	A0A5P8H6S1, serpin-a, *H. longicornis*	0	1727	87.5	45.04 ± 10.59
31	Cl-k.19946 ④	406	Q75Q63, serpin-2 , *H. longicornis*	0	1686	82.4	11.48 ± 2.66
32	Cl-k.16646 ④	201	A0A6M2E637, tick serpins 8, *A. tuberculatum*	7.5E−68	555	63.0	⑻ 178.59 ± 44.58
33	Cl-k.22217 ④	398	A0A023GN51, tick serpins 13, *A. triste*	9.5E−165	1216	61.8	38.49 ± 10.90
34	Cl-k.14429	421	A0A023G8Z1, serine proteinase inhibitor, *A. triste*	0	1409	65.5	71.47 ± 17.26
35	Cl-k.18644 ⑤	229	A0A224YJB7, α2-macroglobulin splicing variant, *Rhipicephalus zambeziensis*	3.7E−141	1119	91.7	⑸ 435.08 ± 100.49
36	Cl-k.18677 ⑤	1142	A0A1E1XEL3, α-macroglobulin, *A. aureolatum*	0	5401	89.6	⑹ 243.02 ± 53.71
37	Cl-k.19779 ⑤	1820	A0A1E1XL07, α-macroglobulin, *A. sculptum*	0	6650	76.1	1.10 ± 0.27
38	Cl-k.18726 et al.	1915	A0A023FNM2, α2-macroglobulin splicing variant, *Amblyomma cajennense*	0	5819	78.9	35.72 ± 8.99
39	Cl-k.18944	269	A0A023GP16, Kazal-type serine protease inhibitor, *A. triste*	5.8E−155	1123	84.0	8.02 ± 1.79
40	Cl-k.12087 ⑥	183	A0A6B9DA14, cystatin, *H. flava*	0	757	100	15.11 ± 4.89
41	Cl-k.17388 ⑥	185	A0A3G6VF56, cystatin, *H. flava*	1.9E−100	745	99.3	65.43 ± 15.54
42	Cl-k.20981 ⑥	164	A0A6M3YRY3, cystatin, *H. flava*	4.2E−89	667	100	15.73 ± 4.93
43	Cl-k.21288	228	A0A023GEH6, thyropin, *A. triste*	1.5E−94	723	60.0	22.19 ± 7.67
44	Cl-k.23450	229	A0A023GAB0, thyropin, *A. triste*	2.2E−89	689	59.4	35.86 ± 8.77
III. Immune-related proteins							
45	Cl-k.18200 ⑦	84	A0A6G5A751, microplusin, *R. microplus*	9.5E−31	260	54.8	⑴ 6514.56 ± 803.43
46	Cl-k.18906 ⑦	134	A0A6G5A751, microplusin, *R. microplus*	8.0E−34	295	52.5	⑷ 699.92 ± 23.27
47	Cl-k.20235 ⑦	87	A0A6G5A751, microplusin, *R. microplus*	2.1E−29	266	46.2	61.60 ± 17.16
48	Cl-k.3924	78	A0A2D1CLH7, defensin DFS2, *H. longicornis*	2.1E−48	361	83.6	11.98 ± 3.53
49	Cl-k.23590 ⑧	1612	A0A131ZDX3, TIL domain-containing protein, *R. appendiculatus*	0	7053	77.0	11.48 ± 2.56
50	Cl-k.13586 ⑧	2610	A0A131Z678, TIL domain-containing protein, *R. appendiculatus*	0	8535	77.2	9.72 ± 2.21
51	Cl-k.18775 ⑧	2252	A0A131YJS1, TIL domain-containing protein, *R. appendiculatus*	0	8966	68.9	1.42 ± 0.30
52	Cl-k.25067 ⑧	109	A0A6M2CNI0,TIL domain-containing protein, *R. microplus*	4.1E−39	329	69.9	26.91 ± 4.60
53	Cl-k.18521 ⑨	235	F0J8I6, ixoderin, *A. variegatum*	5.4E−93	707	66.3	6.23 ± 1.40
54	Cl-k.17959 ⑨	313	A0A1E1XEF5, ixoderin, *A. aureolatum*	5.7E−153	1118	73.8	6.92 ± 1.81
55	Cl-k.19166	177	A0A6M2D1K3, C2b, *R. microplus*	6.9E−55	488	62.3	2.75 ± 0.88
56	Cl-k.21838	428	A0A7L9DI94, C3, *Ixodes ricinus*	1.0E−134	1104	50.9	10.48 ± 2.55
57	Cl-k.14141	152	A0A224Z7V2, serum amyloid A protein, *R. zambeziensis*	1.4E−77	592	72.7	28.19 ± 6.81
58	Cl-k.17842	332	A0A0S3Q1T5, leucine-rich repeat containing protein, *H. longicornis*	0	1464	90.1	19.96 ± 4.17
59	Cl-k.18342	233	A0A0M3TC17, AV422, *H. flava*	8.4E−171	1221	100	12.15 ± 2.84
60	Cl-k.18520 ⑩	181	Q08G07, Hq05, *Haemaphysalis qinghaiensis*	1.1E−129	940	97.2	6.53 ± 1.43
61	Cl-k.18575 ⑩	180	G3BJU6, immunogenic protein, *H. longicornis*	4.5E−120	877	93.9	16.62 ± 4.03
IV. Transporters							
62	Cl-k.25224 ⑪	1538	Q5EG54, Vg, *D. variabilis*	0	5360	67.5	0.21 ± 0.10
63	Cl-k.16576 ⑪	686	E1CAX9, vitellogenin-1, *H. longicornis*	0	1248	98.5	126.50 ± 31.13
64	Cl-k.19213-k18886 ⑪	351	B1B544, vitellogenin-2, *H. longicornis*	8.7E−86	732	98.3	⑶ 1606.10 ± 295.28
65	Cl-k.18067 ⑪	463	B1B544, vitellogenin-2, *H. longicornis*	0	2072	85.1	⑵ 1829.13 ± 312.65
66	Cl-k.19483 ⑪	1386	B1B544, vitellogenin-2, *H. longicornis*	0	4657	62.4	⑽ 138.31 ± 29.98
67	Cl-k.16789 ⑪	114	E1CAY0, vitellogenin-3, *H. longicornis*	3.1E−62	535	85.7	95.40 ± 25.38
68	Cl-k.18851-18114 ⑪	2279	G9M4L6, vitellogenin B, *H. longicornis*	0	13,028	85.9	63.60 ± 12.52
69	Cl-k.21299	207	M5AYG7, ferritin 2, *H. longicornis*	9.9E−133	965	93.5	22.51 ± 4.62
70	Cl-k.19103	152	A0A023G718, fatty acid-binding protein, *A. triste*	4.6E−83	628	85.4	15.26 ± 2.76
V. Muscle proteins							
71	Cl-k.18720	877	J7LVN2, paramyosin, *H. longicornis*	0	4196	98.2	0.53 ± 0.13
72	Cl-k.18460	424	A8E4J9, calreticulin, *H. qinghaiensis*	0	2247	99.5	2.42 ± 0.76
73	Cl-k.18394	52	A0A131ZAE8, tropomyosin, *R. appendiculatus*	0	1423	93.2	3.76 ± 1.07
74	Cl-k.18452	83	A0A0N6X2B1, muscle LIM protein, *H. longicornis*	7.8E−57	417	95.8	4.98 ± 1.14
VI. Heat shock proteins							
75	Cl-k.18505 ⑫	655	A0A097A1J8, heat shock 70 kDa protein 8, *H. flava*	0	3290	100	2.09 ± 0.55
76	Cl-k.18161 ⑫	683	E4W3Z2, heat shock 70 kDa protein 5, *H. longicornis*	0	3317	99.1	0.45 ± 0.22
Other							
Cl-k.18334		255	A0A023FPM9, glycine-rich secreted cement protein, *A. cajennense*	1.1E−118	884	74.0	⑼ 170.44 ± 38.51

Proteins sharing the same number in the Protein ID column belong to the same family. Length indicates the number of amino acid residues of protein fractions detected by MS. The number in the iBAQ column indicates the top 10 most abundant peptides detected by MS

There were many enzymes with anticoagulation activity in tick hemolymph. Among them, serine protease was the most abundant. Three serine proteinase genes from *H. longicornis* (Hl-Sp1, Hl-Sp2, and Hl-Sp3) were cloned, and their recombinant enzymes efficiently hydrolyzed substrates specific for serine proteinases [38]. RNA interference (RNAi) of Hl-Sp1, Hl-Sp2, and Hl-Sp3 genes synchronously caused a decrease in the body weight of engorged ticks, suggesting their synergistic roles in blood-feeding and digestion [38]. Longistatin is an unconventional serine protease that has been shown to hydrolyze fibrinogen and efficiently induce high titers of protective IgG antibodies against ticks [39–41]. Metalloproteinases in tick saliva were found to be essential for blood-feeding [42, 43]. During the initial feeding stage, metalloproteinases suppressed blood clotting and degraded extracellular matrix proteins, which is critical for the preparation of the feeding site. As these enzymes also demonstrated anti-angiogenic activity, they were of importance in the late feeding stage by inhibiting tissue repair in the host. *Rhipicephalus microplus* secreted carboxylic ester hydrolase in the skin of calves, immediately adjacent to mouthparts, or in the attachment cone [44]. This constitutes an enzyme system against coagulation together with serine protease and metalloproteinases, among others.

Enzymes in tick hemolymph also take part in nutrient metabolism. Aspartic and cysteine proteinases and exopeptidases were shown to catalyze the decomposition of Vg and Hb [45, 46]. Cathepsin L-like cysteine endopeptidase was reported to hydrolyze synthetic substrates and protein substrates including Hb [47, 48], and serine carboxypeptidase and cathepsin C broke down small peptides, releasing free amino acids [46, 49]. Glutathione S-transferases facilitated the excretion of physiological and xenobiotic substances, protecting cells against chemical toxicity and stress [50]. Although specific roles of glyceraldehyde-3-phosphate dehydrogenase and fructose-1,6-bisphosphate aldolase have not been verified in ticks, they are key enzymes in carbohydrate metabolism.

Some enzymes have appeared in hemolymph with innate immune activity. Liao et al. cloned genes encoding putative protein disulfide isomerase (Hl-PDI1, Hl-PDI2, Hl-PDI3), lysozyme (Hl-lysozyme), and lysosomal acid phosphatase (HL-3) in *H. longicornis* ticks. Hl-PDI1/2/3 were expressed in all developmental stages and in organs including the midgut, salivary gland, ovary, hemolymph, and fat body of adult females, and Hl-PDI1/3 was possibly involved in *Babesia* infection [13]. Increased gene expression of Hl-Lysozyme was observed in female ticks challenged with bacteria, implying a possible role in the innate immunity of ticks against microorganisms [51]. HL-3 transcripts were significantly induced by blood-feeding, and were involved in tick innate immunity [52]. Superoxide dismutase (SOD) was reported as a key enzyme in detoxification of reactive oxygen species, and silencing of Cu/Zn-SOD decreased the colonization of *O parkeri* in *A. maculatum* ticks [53].

We also detected chitinase in *H. flava* hemolymph. Chitinase was induced by ecdysteroids to degrade older chitin at the time of molting, and recombinant chitinase from *H. longicornis* was capable of chitin degradation [54, 55].

### Protease inhibitors in tick hemolymph

Numerous protease inhibitors have been detected in tick hemolymph, including serine protease inhibitors, tight-binding inhibitors, cystatins, and thyropins.

α-2-macroglobulin, serpins, and Kunitz/Kazal domain-containing proteins were found to belong to the serine protease inhibitors [56]. Serpins were the most abundant protease inhibitors in *H. flava* hemolymph. In total, about 120 serpins were recorded in ticks [57–59]; 20 of them in different tick genera have been functionally characterized [60]. A serpin from *Rhipicephalus haemaphysaloides* was shown to participate in vitellogenesis [61]. Additionally, serpin-2 and other serpins were directly related to tick blood-feeding, facilitating successful acquisition of a blood meal [62–64].

Cystatins and thyropins were found to be inhibitors of cysteine peptidases. Tick cystatins either regulated cathepsin-mediated Hb digestion [46] or played a role in tick embryogenesis [65]. In addition to these functions, a type-2 cystatin in the hemocytes of *R. microplus* was related to tick immunity [66].

### Immune-related proteins in tick hemolymph

Three microplusins were detected. They were 103 amino acids in length; all contained signal peptides. They displayed similarity of 46.2–52.5% compared with a microplusin from *R. microplus* [67]. Microplusin was shown to have bacteriostasis activity (gram-positive bacterium) and to offer protection against *Rickettsia rickettsii* infection [67, 68]. Microplusin gene expression was verified in several organs, including fat body, hemocyte, ovary, and midgut [67, 68]. A microplusin-like peptide was identified in *A. hebraeum* hemolymph [69].

### Transporters in tick hemolymph

There were three types of transporters in tick hemolymph, i.e., Vg, ferritin, and fatty acid-binding protein. The Vg family contained the most members. Vgs have been investigated and partially characterized in *O. moubata* [4, 70], *O. parkeri* [29], *D. variabilis* [31, 71], *I. scapularis* [3], *H. longicornis* [33], and *R*. *microplus* [72], among others. Vg is synthesized in fat bodies, gut cells, and, to a lesser extent, ovaries of tick females after mating and feeding [2]. Based on the unique peptides obtained, a minimum of seven types of Vgs were retrieved in *H. flava*. Cl-k.16576, Cl-k.19213, and Cl-k.16789 displayed a notable similarity with Hl-Vg1, Hl-Vg2, and Hl-Vg3, respectively. Hl-Vgs consisted of four major polypeptides [33]. Hl-Vg RNAi-challenged ticks displayed lower body weight and egg weight and higher mortality in engorged females [33]. Cl-k.25224 was structurally similar to Vg in *D. variabilis* (UniProt accession number: Q5EG54, GenBank accession number: AY885250). Vg mRNA was detected in replete (mated) pre-ovipositional female *D. variabilis*, increased to a more notable level in ovipositing females, and was absent after completion of egg-laying [31].

### Muscle proteins and heat shock proteins in tick hemolymph

There were four muscle proteins detected in tick hemolymph, including paramyosin, calreticulin, tropomyosin, and muscle LIM protein. Aside from muscle composition, they demonstrated other special functions. For example, the recombinant *B. microplus* paramyosin was able to bind both IgG and collagen [73], while calreticulin from *A. americanum* was found to bind to C1q [74]. Silencing of *H. longicornis* tropomyosin (HL-Tm) led to a reduction in tick engorgement and oviposition [75].

Two heat shock proteins 70 (HSP70) were found in tick hemolymph, and their expression was significantly upregulated upon blood-feeding [76, 77]. HSP70-8 and HSC70 were shown to exert an anticoagulation effect in vitro [78].

### Quantitative analysis of proteins in tick hemolymph

The iBAQ of high-confidence host proteins in *H. flava* hemolymph is listed in Table 1. The top 10 abundant host-derived proteins included Hb subunit-α and subunit-β, albumin, serotransferrin-like, ubiquitin-like, haptoglobin, α-1-antitrypsin-like protein, histone H2B, apolipoprotein A-I, and C3-β.

Vitellogenin, microplusin and α-macroglobulin were the top three abundant tick proteins (Table 2). The abundance of Vg1, Vg2 and Vg3 was extremely high in the hemolymph of *H. longicornis* [33]. Our unpublished data indicate that the egg protoplasm did not contain large quantities of these Vgs, implying that the main role of these Vgs might not be as nutrients. The high abundance of Vg and microplusin indicated that the major function of tick hemolymph was the transport of substances and participation in the immune responses.

Of note, Cl-k.18334, which was annotated as a glycine-rich secreted cement protein (A0A023FPM9), was ranked as the ninth most abundant tick-derived protein in hemolymph. Thus far, there have been no reports on its function in ticks.

### Protein families in tick hemolymph

Twelve protein families were identified in this study: serine protease, carboxylic ester hydrolase, chitinase, serpin, α-macroglobulin, cystatin, microplusin, TIL domain-containing protein, ixoderin, immunogenic protein, Vg, and heat shock protein. However, only four families, namely, serpin, Vg, cystatin, and microplusin, have been investigated extensively in ticks.

Sequence analysis revealed that some families had extremely similar sequences among members. For instance, three microplusins shared up to 86.22% sequence similarity (Additional file 2: Fig. S1). All had an N-terminal sequence MKA, six C residues, and signal peptides.

Proteins in some families, such as cystatin and serpin, shared remarkable similarity in structure, although their amino acid sequences were quite different. Cl-k.17388, Cl-k.20981, and Cl-k.12087 were all cystatins; the similarity between them was 35.92%. However, both had conserved a GG at the N-terminal, and a QXVXG motif of cystatin2 and a typical C-PW-C motif at the C-terminal (Additional file 3: Fig. S2).

Seven serpins all contained serpin consensus amino acid motif N-[AT]-[VIM]-[YLH]-F-[KRT]-[GS], [DERQ]-[VL]-[NDS]-E-[EVDKQ]-G, and serpin signature PS00284 ([LIVMFY]-[G]-[LIVMFYAC]-[DNQ]-[rkHQs]-[PST]-F-[LIVMFY]-[LIVMFYC]-X-[LIVMFAH]).

Among the four TIL domain-containing proteins, Cl-k.25067 shared similarity with ixodidin, an antimicrobial peptide from hemocytes of *R. microplus* with inhibitory activity against serine proteinases [79]; the other three TIL domain-containing proteins (Cl-k.23590, Cl-k.13586, and Cl-k.18775) all included a trypsin inhibitor-like, cysteine-rich domain and a von Willebrand factor type domain in their structures, and might play a role in hemolymph anticoagulation [80].

Importantly, the present study only provided a protein profile in the hemolymph of fully engorged ticks at a single time point in blood-feeding. Further studies will address the importance of hemolymph proteins during feeding, and will include the unfed tick stage and different time points.

## Conclusion

Based on a search against the UniProt Erinaceidae database and *H. flava* proteome library, we identified 18 host-derived high-confidence proteins and 169 tick-derived high-confidence proteins, providing the most comprehensive protein composition in tick hemolymph thus far. The protein profile of the *H. flava* hemolymph mirrored a sophisticated protein system in the physiological processes of anticoagulation, blood meal digestion, and innate immunity. As the bulk of proteins detected in hemolymph have not been functionally characterized in ticks, further investigations are needed to decipher their roles in tick biology.

## Supplementary Information

**Additional file 1: Table S1.** Unique peptides and deduced protein sequences identified by searching against the *H. flava* transcriptome database.

**Additional file 2: Figure S1.** Amino acid sequences of three microplusins in *H. flava* hemolymph.

**Additional file 3: Figure S2.** Amino acid sequences and domains of two cystatins in *H. flava* hemolymph.

## Data Availability

The data supporting the conclusions of this article are available in the iProX repository, https://www.iprox.cn/page/DSV021.html;?url=1642501651308hONl, with the key 3ABj.

## Abbreviations

Hb: Hemoglobin
iBAQ: Intensity-based absolute quantification
LC–MS/MS: Liquid chromatography–tandem mass spectrometry
MS: Mass spectrometry
SDS-PAGE: Sodium dodecyl sulfate–polyacrylamide gel electrophoresis
Serine proteinase inhibitor: Serpin
Vg: Vitellogenin
