# Supplementary figures and images for: Protein profiling of hemolymph in Haemaphysalis flava ticks
Source: Parasit Vectors. 2022 May 24;15:179. doi: 10.1186/s13071-022-05287-7 (PMC9128142; doi:10.1186/s13071-022-05287-7)

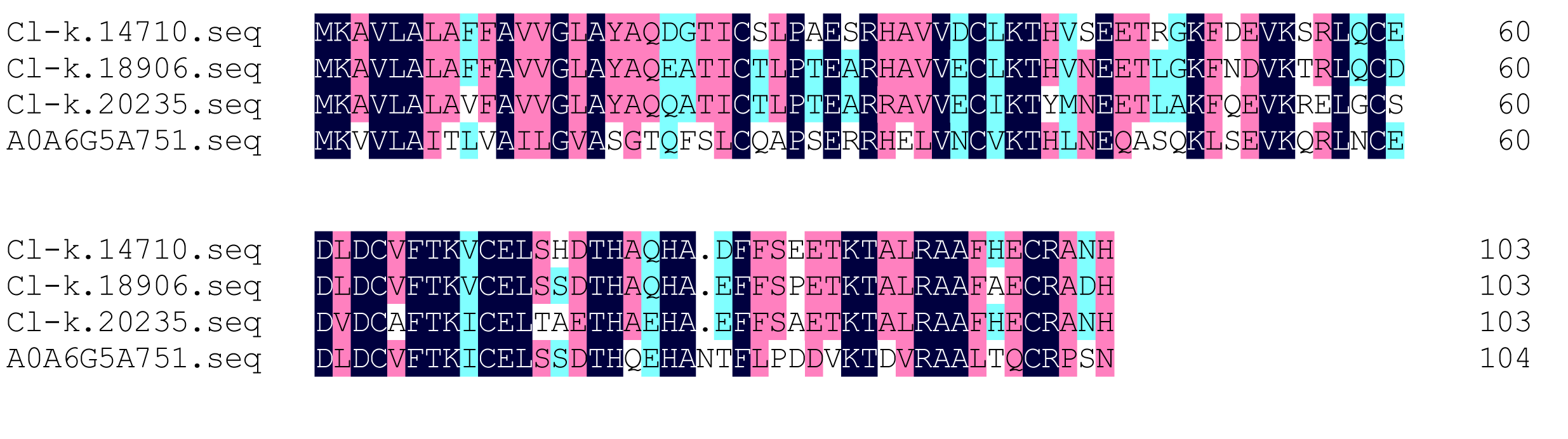

Supplement: Supplementary file 2 — Additional file 2: Figure S1. Amino acid sequences of three microplusins in H. flava hemolymph. [file 13071_2022_5287_MOESM2_ESM.tif]

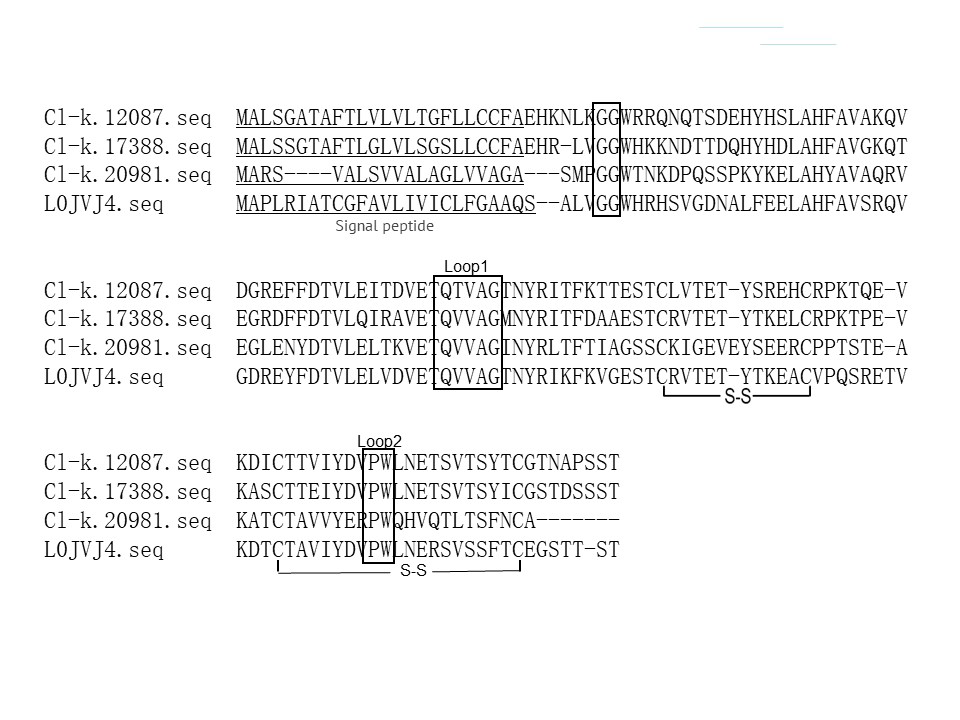

Supplement: Supplementary file 3 — Additional file 3: Figure S2. Amino acid sequences and domains of two cystatins in H. flava hemolymph. [file 13071_2022_5287_MOESM3_ESM.tif]
